# Talent acquisition and technology: A step towards sustainable development

**DOI:** 10.3389/fpsyg.2022.979991

**Published:** 2022-10-18

**Authors:** Saqib Rehman, Aman Ullah, Khalida Naseem, Ali Raza Elahi, Humaira Erum

**Affiliations:** ^1^Department of Management Sciences, Lahore College for Women University, Lahore, Pakistan; ^2^UVAS Business School, University of Veterinary and Animal Sciences, Lahore, Pakistan; ^3^Department of Management and Marketing, Faculty of Business and Economics, University of Melbourne, Melbourne, VIC, Australia; ^4^Faculty of Economics and Management Science, School of Business Management, Minhaj University, Lahore, Pakistan; ^5^Department of Commerce and Finance, Government College University, Lahore, Pakistan; ^6^Department of Management Sciences, National University of Modern Languages, Lahore, Pakistan

**Keywords:** social media recruiting technology, recruitment, UTAUT2, information technology, human resource management, sustainable development

## Abstract

**Purpose:**

The purpose of the research is to highlight the use of social media and information technology in employee recruitment by providing a conceptual recruitment model in the aspects of theoretical foundations and empirical evidence on the factors and outcomes leading to the use of social media recruiting technology (SMART).

**Design/methodology/approach:**

A total of 4,481 organizations are listed as the population of the study, and a total of 589 responses are used in the study for data analysis. Selection of the sample is done based on the simple random sampling technique. Appropriateness of sample size is confirmed with the help of G*Power (3.1.9.2) software, which calculated a sample size of 326 with 99% power, a multiple correlation (R) of 0.30, and at a significance level of 0.05.

**Findings:**

The paper provides empirical insights into the elements of the extended unified theory of acceptance and use of technology; i.e., performance expectancy, effort expectancy, social influence, facilitating conditions, hedonic motivation, habit, and price value have positive relations with the intention to adopt social media recruiting technology, and the intention impacts use of social media recruiting technology. Furthermore, the use of social media recruiting technology impacts outcome variables, i.e., social media recruiting time, cost, and recruitment quality, which establish the extension of the recruitment model with all factors and outcomes.

**Originality:**

This study provides a model of employee recruitment to win the battle of acquiring human capital using social media recruiting technology as a step toward sustainable development, which has been conceptually defined and empirically tested. The comprehensiveness of the model has never been discussed in earlier studies.

**Practical implications:**

Through this research, organizations will get an opportunity to experience enhancement in the scope of recruitment as a whole instead of considering recruitment as a traditional procedure, and the use of information technology can be expanded for progressive measures for future purposes and pandemic situations.

## Introduction

To attract the pool of potential candidates, the organizations preferred to utilize the mediums such as television advertisements (Taylor and Schmidt, 1983), newspapers (Breaugh, 1981; Jones et al., 2006), radio (Saks, 1994), web advertisements (Llorens and Kellough, 2007), and outsourcing (Ordanini and Silvestri, 2008; Shih and Chiang, 2011). The organization’s employee recruitment process has undergone significant transformations in the recent years. Across the globe, recruitment departments of the organizations have employed electronic channels, which are exclusively asserted by an en route progression of technologies (Bondarouk et al., 2017; Vetráková et al., 2018; Van Esch et al., 2019). Keeping the breast up with changing world scenarios and dealing with the prevailing situation of the current pandemic, the organizations have realized the need and use of social networking websites (SNWs) for recruiting top and rare talent to achieve innovation and sustainability. SNWs have given a new face to recruitment trends (Villeda et al., 2019; Nikolaou, 2021; Rehman et al., 2021). Social networking websites are no longer just a place to keep in touch with social circles, but they have entered the workplaces (Chang et al., 2022) in the form of social media recruiting technology (SMART) and becoming a standard mode of employee recruitment and selection.

Recruitment managers in the organizations are now experiencing advancements in recruitment using SNWs such as LinkedIn, Facebook, Twitter, YouTube, Xing, and Plaxo (Chua et al., 2018; Golovko and Schumann, 2019). These websites provide a user-friendly interface through the interaction of humans and information technology (Suen, 2018). SNWs are the authentic and reliable medium for talent hunting and have become a comprehensive source of hiring decisions. Equally, SNWs use specialized algorithms to provide convenient recruitment processes and help organizations reach sustainability. Prior literature (Toft et al., 2014; Peñarroja et al., 2019; Rehman et al., 2021) suggested that social networking websites help in the advertisement for a job, targeting and creating a larger pool, screening potential applicants, talent hunting based on specific organizational criteria, and focusing on specific skills required for hiring decisions. However, there is a knowledge gap: “Which pre-hire recruitment objectives can be achieved using SNWs?” Therefore, the use of SNWs in organizations must be explored further to develop a comprehensive recruitment model for devising better strategies. Hence, it is imperative to seek theoretical and practical corroboration on the feasibility of the social media recruiting technology (SMART) use decision and how this technology can help to achieve desired recruiting outcomes of an organization for organizational sustainability.

The primary purpose of this research was to highlight the use of social media and information technology in employee recruitment by providing a comprehensive recruitment model in the aspects of theoretical foundations and empirical evidence on the factors and outcomes leading to the use of social media recruiting technology. There are two main reasons for the motivation to focus on Pakistani (Asian) organizations. First, according to the figures from Internet World Stats (2021), Pakistan has witnessed a significant and rapid increase in internet use (current users: 100,679,752) compared to other countries. Similarly, the number of users of social media websites has also increased, and approximately 43.55 million users use the Facebook platform only (Statista, 2022). Second, although the number of social media users has increased, the use of social networking websites in hiring employees is still the less studied area, especially regarding its outcomes such as time, cost, and recruitment quality.

The findings can support Pakistani organizations and relevant stakeholders in devising better strategies to use social networking websites for hiring, leading to employee retention for organizational sustainability. Through this research, organizations will get an opportunity to experience enhancement in the scope of recruitment as a whole instead of considering recruitment as a traditional procedure and the expansion in the use of information technology for future purposes and pandemic situations. Also, the potential candidates and employees excessively use several social networking websites, which can be an opportunity for further enhancing understanding and use of social networking websites because they can investigate the relationship between SNWs and post-hire recruitment outcomes. The current study will help managers modify traditional recruitment through social media and technological advancements. In conclusion, a model of social media recruiting technology for employee recruitment has been validated in the contextual framework of Pakistan (Asian) to fill the theoretical and empirical gap in the current body of knowledge.

## Background literature and hypothesis development

Social networking websites (SNWs) are considered a vital recruitment tool though recruiting through social media technology is an ongoing credible area of concern for researchers and practitioners (Villeda et al., 2019; Dwivedi et al., 2020). Prior research raised issues such as technological advancements in employee recruitment requiring organizations to allocate additional assets, administrative support, and substantial changes in existing recruitment methods (Ellabban and Abu-Rub, 2016). Similarly, anecdotal shreds of evidence were presented in the business context but only in periodicals and industry reports (Kashi, 2015; Rehman, 2018). A little empirical and hypotheses-driven research has been done to understand the prevailing advancements regarding using SNWS and incorporating technology in recruitment (Dwivedi et al., 2020). Therefore, a model of employee recruitment using social media recruiting technology (SMART) has been conceptually defined and empirically tested in this study as a step toward sustainable development. The current study used the Extended Unified Theory of Acceptance and Use of Technology (UTAUT2) (Venkatesh et al., 2012) as the underpinning theory in the use of SMART and for the evaluation of pre-hire outcomes of SMART in an organizational setting.

The Extended Unified Theory of Acceptance and Use of Technology is the most widely-used model in the technological community and is a refinement of earlier models. This model attempts to centralize previously scattered research on end-users’ use of technological change into a coherent framework. There are many reasons why the extended UTAUT (Venkatesh et al., 2012) should be used as the appropriate model for SMART adoption. The utility of UTAUT2 lies in its ability to foresee people’s intent to adopt new technologies based on its emphasis on utilitarian value, which is inextricably related to extrinsic motivation. In addition to the original four constructs of UTAUT, the extended version, known as UTAUT2, added hedonic motivation, price value, and habit as key predictors to measure intention. Hedonic motivation refers to the pleasures of using technology in the workplace, whereas price value refers to the financial investment required to acquire and set up new technologies. One final component in UTAUT2 that shapes a manager’s decision to embrace SMART is a habit.

### Relationship between extended unified theory of acceptance and use of technology factors and intention to adopt social media recruiting technology

After reviewing the relevant literature on social media recruiting technology, it has been observed that the intervention of technology concept has gained much scholarly attention in recent years. In the study of Barba-Sánchez et al. (2021), the model illustrates the connection between a secondary school’s technological aptitude and a smart city’s commercial activities. The study also demonstrates a favorable correlation between secondary schools’ IT capabilities and the entrepreneurial activity in the city. Similarly, the new production model appears to depend heavily on managing information and knowledge in an environment of economic instability and intense competition between businesses. Corporate performance is significantly impacted by a firm’s adoption of information and communications technology (ICT) and industry attractiveness. This research recommends complete ICT integration inside businesses (Barba-Sanchez et al., 2018). Prior studies (Kashi, 2015; Nikolaou, 2021; Rehman et al., 2021) highlighted the seven factors influencing the intention to adopt social media recruiting technology.

#### Performance expectancy

Performance expectancy can be defined as a level of expectation an individual may have from technology that the utilization of technology will assist him in enhancing the level of performance at a job (Kemp et al., 2019). Venkatesh et al. (2003) discussed performance expectancy as the degree of belief an individual may have that using a system will help an individual to achieve job outcomes for increasing organizational performance. The expected outcomes of high performance from technology will be a driving source of intention for an individual to use technology. As per the different models mentioned above, performance expectancy is the most substantial factor influencing the intention to utilize technological means (Alalwan et al., 2018). When Venkatesh et al. (2003) removed two essential factors of the technology adoption model, i.e., performance expectancy and effort expectancy, to see the impact of the remaining factors, the results were diminished. Therefore, performance expectancy will represent the degree to which an individual believes that using technology and social networking websites in recruitment will positively impact his job performance. As per the above literature, the following hypothesis has been proposed in the current research.

*H1a: Performance expectancy positively relates to the intention to adopt social media recruiting technology.*

#### Effort expectancy

The concept of effort expectancy can be defined as the degree to observe an association between conveniences of the utilization of the system (Bhatiasevi, 2016). The convenience of using technology has a positive relationship with the intention of technology adoption as if someone believes that he has the skills and competency to use technology, then there is a high probability of accepting technology utilization (Kurfalı et al., 2017; Nahla Aljojo, 2020). Venkatesh et al. (2003) have discussed effort expectancy as the most important factor along with performance expectancy, which significantly impacts the intention to adopt the technology. Thus, effort expectancy denotes individuals who believe that accepting social media in the recruitment process is not a rigid or multifarious procedure and that utilizing this technology will positively impact their job performance. As per the above literature, the following hypothesis has also been proposed.

*H1b: Effort expectancy positively relates to the intention to adopt social media recruiting technology.*

#### Social influence

According to Venkatesh et al. (2003), social influence plays a vital role in developing the intention to adopt technology and make decisions for an individual. Gershman et al. (2017) and Rehman et al. (2017) highlighted that there are some significant factors present in individuals’ social systems that shove them to decide their influence and it can be about adopting some technology. Social influence in technology adoption can be defined as the ability to perceive that people important to him believe he should utilize and adopt the technology. In the initial phase of SMART adoption, social influence in the variable is known to impact the individual’s intention significantly, strengthening the relationship between these two constructs. If someone around is giving importance and significance to work or the use of technology, then there is a likelihood of the individual being more inclined toward the work (Rehman et al., 2017) and technology adoption (Ifinedo, 2016). Thus, under this research, social influence is the degree to which an individual perceives that people around him believe that he should accept the use of social media and technology in the context of recruitment. As per the above literature, the following hypothesis has also been proposed.

*H1c: Social influence positively relates to the intention to adopt social media recruiting technology.*

#### Facilitating conditions

The construct of facilitating condition can be defined as the degree to which an individual has faith that organizational and technical infrastructures positively impact the system’s utilization (Venkatesh et al., 2003). The facilitating conditions include human resources, an existing system with adequate technical competency, internal and external support, financial strength, and training and development programs to enhance the skills and competencies. Upon analyzing when the individual believes that infrastructure supports the use of the system, he/she is more likely to adopt the technology (Oliveira et al., 2016; Macedo, 2017). Under this research, the variable of facilitating condition is considered as the degree to which an individual believes that the organizational infrastructure supports financial, human, technical, and intellectual resources along with the presence of technological infrastructure that will support the use of social media and technology in the context of the recruitment process. As per the above literature, the following hypothesis has also been proposed.

*H1d: Facilitating conditions positively relate to the intention to adopt social media recruiting technology.*

#### Hedonic motivation

For the understanding of hedonic motivation, Sitar-Tãut (2021) performed an empirical investigation regarding mobile learning use in the social distancing of COVID-19 and highlighted its mediation role. Research on hedonic motivation relates it to the fun factor achieved through individuals’ technology experiences (Baabdullah, 2018; Tamilmani et al., 2019; Al-Azawei and Alowayr, 2020). Thus, hedonic motivation is considered one of the significant factors that impact the intention of an individual to adopt a technology. Using this construct is integrated with the fun side of using technology, and considering work as enjoyment can positively influence SMART adoption. In the context of technology adoption, hedonic motivation serves as the joy and fun elements derived from technology utilization, which is evidence that hedonic motivation plays a vital role in the use and adoption of technology in the recruitment process. Thus, in this research, the construct of hedonic motivation represents the degree to which an individual perceives using and adopting social media recruiting technology, which will be fun and joy during the recruitment process. As per the above literature, the following hypothesis has also been proposed in the current research.

*H1e: Hedonic motivation positively relates to the intention to adopt social media recruiting technology.*

#### Habit

In the context of behavioral intention, habit is known to have a direct or indirect impact on behavioral intentions (Baabdullah, 2018). A habit of using technology can be defined as an automated process that comes into its realization when an individual experiences using technology time and again. An individual needs to have an experience of using technology, but it is not enough to develop a habit; thus, the habit is developed when an individual explores and experiences the technological features in multiple periods (Morosan and DeFranco, 2016; Tamilmani et al., 2018). Thus, the habit variable can be positively related to social media recruiting technology adoption by considering the impact of previous learning experience and know-how about technology. Venkatesh et al. (2012) stated habit as a construct in which individuals are automatically bound to behave in a certain way due to learning. As per the above literature, the following hypothesis has also been proposed.

*H1f: Habit positively relates to the intention to adopt social media recruiting technology.*

#### Price value

Cost connected with the use of technology is an essential factor. Venkatesh et al. (2012) defined price value as a tradeoff between the perceived advantages associated with technology and the cost required to adopt a technology. This tradeoff between benefits and cost has a significant influence on developing consumer’s intention about using technology. On the one side, the consumer spends costs for adopting new technology; on the other side, there are so many financial paybacks that consumers will acquire from using technology (Faqih and Jaradat, 2021). Therefore, the perceived value of the technology is directly proportional to the cost incurred and other financial benefits gained after its adoption (Merhi et al., 2019). Organizations consider technology necessary if the benefits are improved efficiency, time-saving, and cost-effectiveness (Chen et al., 2021).

The literature has also highlighted some contrary results that perceived higher cost of technology adoption might negatively influence the consumer’s intention (Verkijika, 2018; Beh et al., 2021). Factors that can convert this negative influence into positive are the perceived associated benefits of the technology (Tamilmani et al., 2021). As per the above literature, the following hypothesis has been proposed.

*H1g: Price value positively relates to the intention to adopt social media recruiting technology.*

### Intention to adopt social media recruiting technology and use of social media recruiting technology

The intention of technology adoption can be defined as an individual considering to assume an act that leads to a prediction of similar behaviors when individual acts as per his willingness (Al-Okaily et al., 2020). The intended behavior can be known as the motivational factor that impacts behavior and determines the potential indicators of an individual’s willingness and also defines the level of effort to engage in a particular act or behavior (Parry and Wilson, 2009; Li et al., 2021). Thus, the intention to adopt social media recruiting technology is the main factor that impacts an organization’s decision to utilize technological or innovative systems.

In UTAUT2, behavior is the outcome one expects after implementing the theoretical model and concepts, whereas the consideration of the use of SMART is a behavioral factor in the model. The use of SMART can be denoted as the implementation and use of technology for employee recruitment processes, which is led by the intentional behavior of SMART adoption (Olasina and Mutula, 2015). As per Venkatesh et al. (2012), the use of technology includes the strength, intensity, and frequency of technology used for some purposes in the companies; thus, Kashi (2015) mentioned the use of technology as gathering a pool of potential candidates by attracting, sourcing, and approaching them through an initial contact list for a specific job role with making effective use of social networking websites. As per the above literature, the following hypothesis has also been proposed in the current research.

*H2: Intention to adopt social media recruiting technology is positively related to the use of social media recruiting technology.*

### Use of social media recruiting technology and social media recruiting technology cost

A recruitment process model by Breaugh (2008) suggested that aligning and devising a suitable recruitment process is similar to achieving recruitment goals. Several researchers stated that the objectives or outcomes of recruitment could be further segregated into two dimensions, i.e., pre-hire and post-hire objectives (Breaugh, 2008, 2017; Kashi, 2015; Rehman, 2018; Pessach et al., 2020). Extensive research has been conducted in this perspective and pondered multiple features of post-hire outcomes, whereas the amount of information and research on the technological use aspect of pre-hire outcomes is less. The post-hire objectives include a low rate of turnover, positive retention rate, increased job satisfaction, employee engagement, and employee loyalty toward the company, and these variables have been measured and evaluated by the researcher repeatedly. Thus, another aspect of this research is to scrutinize pre-hire outcomes after accepting social media recruiting technology in the recruitment process.

Research about pre-hire recruitment outcomes using social media is still in its earlier phases. However, for the current aspect of the study, three outcomes of the use of SMART are highlighted through literature, i.e., SMART Cost, SMART Time, and SMART Recruitment Quality, whereas for pre-hire objectives, extensive research conducted by Kashi (2015) has been used and all definitions are extracted as per need and criteria. SMART cost is the conception of an individual in the association of cost with attracting, sourcing, and hiring candidates. The cost incurred for recruiting is one of the significant factors in HRM that affects the usage of technology; as for organizations, the use of technology is highly relevant to reducing costs in HR operations (Alsultanny and Alotaibi, 2015; Okolie and Irabor, 2017). Geyskens et al. (2006) highlighted that organizations constantly strive to minimize the cost of operations, and use of social media recruiting technology can reduce the overall cost of employee recruitment. Finding the right employee for the right position at the lowest possible cost is challenging for the organization (Melanthiou et al., 2015). Therefore, the high cost to perform the recruitment activity through traditional ways may be the encouraging or relative advantage that organizations may seek from SMART where this cost is comparatively lower. By considering the literature discussed, the SMART cost can be taken as one of the pre-hire outcomes of the use of SMART, and the following hypothesis can be proposed in the current research.

*H3: Use of social media recruiting technology is positively related to SMART cost.*

### Use of social media recruiting technology and social media recruiting technology time

By referring to the pre-hire objectives, employee recruitment is attracting, screening, selecting, and hiring the best among a larger pool of quality candidates for finding the person-organization fit. This activity, which comprises many hierarchal steps, takes extensive time and cost for successful completion; therefore, the traditional employee recruitment process is more time-consuming than social networking websites (Melanthiou et al., 2015). Other than the more extended period taken by the usual hiring process, authenticating the information provided by the candidates is also one of the biggest challenges for the recruiters (Ullah and Rehman, 2018). To improve the time required for the hiring process, organizations shifted toward online recruitment channels, and nowadays the best form is hiring through SNWs (Jin et al., 2016; Sengupta et al., 2021). The way organizations hire employees is changing quickly, and the tools developed for this purpose have evolved rapidly (Claus, 2019). It is a clear shift toward innovation and modernization (Kashi, 2015); therefore, SMART time can be described as a conception of an individual considering association of time required for the fulfillment of a vacant job position.

Considering the literature discussed, SMART time can be taken as one of the pre-hire outcomes of the use of SMART, and the following hypothesis can be proposed in the current research.

*H4: Use of social media recruiting technology is positively related to SMART time.*

### Use of social media recruiting technology and social media recruiting technology recruitment quality

It has become the norm for recruiters to believe that using social networking websites for hiring purposes enables better and quality candidates (Kashi, 2015; Ruparel et al., 2020). Through SNWs, quality candidates can be targeted by identifying the required knowledge, skills, and abilities. Furthermore, using the right social media platform is crucial as it dramatically affects recruitment quality and the number of applications received through used media (Villeda et al., 2019). Selection of the wrong platform to communicate and process job openings could result in receiving massive or unrelated applicants in the form of negligent hiring, or this situation can be vice versa. Therefore, recruiters seemed to comprehend these facts and were inclined to accept social media technology to search for and recruit new candidates (Alexander et al., 2019). SNWs enable recruiters to set out their recruitment strategies to access quality recruits, and their management has improved using these platforms during the hiring process (Manuaba and Darma, 2021). An organization that accepts the social media recruiting technology will be in a position to attract potential passive candidates who might not apply for an advertised vacancy otherwise, and it also helps in getting more statistics about the candidates for assessing them better earlier to take final hiring decision (Manuaba and Darma, 2021; Wenzhi et al., 2021). SMART recruitment quality can be described as the quality and potential of human capital that can be accessed in the shape of a larger pool through social media recruiting technology.

Considering the literature discussed, SMART recruitment quality can be considered one of the pre-hire outcomes of use of SMART, and the following hypothesis can be proposed in the current research.

*H5: Use of social media recruiting technology is positively related to SMART recruitment quality.*

### A conceptual framework

**FIGURE 1:**
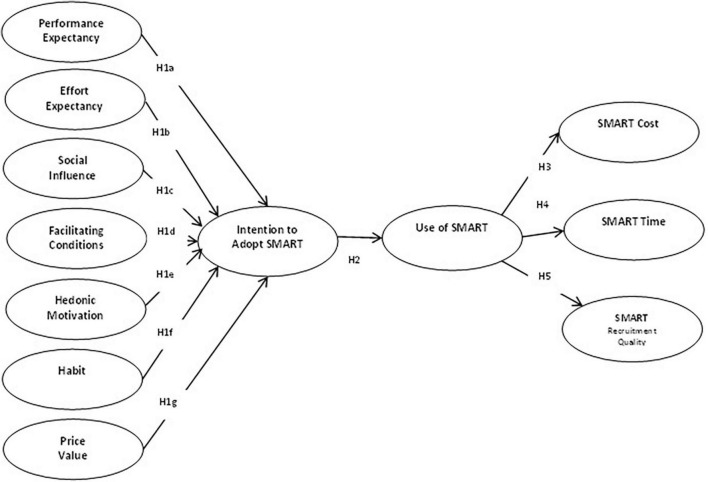
Research model and proposed hypotheses.

Based on the review of prior literature, the current study developed the conceptual framework and proposed hypotheses (refer to Figure 1).

## Research methodology

### Sample and procedure

The primary respondents of the study were HR managers or managers involved in recruitment activities of different organizations in Pakistan. For the generalizability of results, organizations operating in four diverse industries were taken, and they included manufacturing, services, exports, and trades. These organizations were listed with the Pakistan Stock Exchange (PSX) and the Lahore Chamber of Commerce. A total of 4,481 organizations were listed as the population of the study and a total of 589 responses were used in the study for data analysis. The selection of the sample was done based on the simple random sampling technique. Appropriateness of the sample size was confirmed with the help of G*Power (3.1.9.2) software, which calculated a sample size of 326 with 99% power, multiple correlations (R) of 0.30, and a significance level of 0.05. Another confirmation of sample size was acquired from the study of Johnson and Gill (2010) and Taherdoost (2017), which described the sample size as 357 when the population size is 5,000 with a variance of population of 50%, a confidence level of 95%, and a margin of error of 5%. Data analysis was performed using SPSS and AMOS version 20.

In Table 1, demographic information is shown, revealing that male respondents (83.4%) are more in numbers than female respondents (16.1%). In total, 3% of the participants belonged to the age group of 18–25, whereas 39% of the respondents were under the age group of 26–35, respondents belonging to the age group of 36–45 were 45%, 12% belonged to the age group of 46–55, and the 2% above 55 years. Therefore, we can say that majority of the respondents belong to the middle age of 26–45. Respondents worked at different positions but were involved in recruitment decisions, and 1% of the respondents’ profiles belonged to the post of CEO and general managers, 32% of respondent profiles were of managers, 65% of the profiles were of HRM persons/heads, and only 1% of the respondents were from different departments. In total, 12.7% of the organizations approached had an employee population less than 100, 36% of companies had an employee population ranging from 101 to 200, 26.5% of companies had an employee population ranging from 201 to 300, 24.3% of companies had an employee population ranging from 301 to 500, and less than 1% companies had an employee population of above 500. Data were collected through the manufacturing and service industries, of which 47% were from manufacturing organizations and 53% were from the service sector.

**TABLE 1:** Demographic attributes.

Demographic characteristics		*n* = 589	%
Gender of recruiting managers	Male	494	83.4
	Female	095	16.1
Age of recruiting manager (in years)	18–25	017	2.9
	26–35	230	39
	36–45	264	44.8
	46–55	070	11.9
	55 above	012	02
Designation	CEO	003	0.5
	General managers	005	0.8
	HRM heads	191	32.4
	Recruitment managers/HR head	384	65.2
	Others	006	1
Size of organization	Less than 100	075	12.7
	101–200	214	36.3
	201–300	156	26.5
	301–500	143	24.3
	501 and above	001	0.2
Operating sectors	Services	311	52.8
	Manufacturing	278	47.2

### Measures of the constructs

The established measures were adopted in the study, and all used items were measured using a seven-point Likert scale. An extensive literature review was conducted to develop the questionnaire based on pre-existing measures. For the face validity of the questionnaire, three Ph.D. experts in the field of human resource management and five HR managers were contacted to review and suggest possible changes if required. Minor changes were made to the survey questionnaire based on the suggestions of experts and managers. The measures and the items of each variable in the current study are mentioned in Table 2.

**TABLE 2:** Constructs and items used in the study.

Construct and references	Items
Performance expectancy Venkatesh et al. (2012)	PE1. Recruitment staff find social networking websites useful in their jobs.
	PE2. Using social networking websites increase the chances of successful recruitment.
	PE3. Social networking websites help in accomplishing recruitment-related things more quickly.
	PE4. Using social networking websites increase the productivity of recruitment staff.
Effort expectancy Venkatesh et al. (2012)	EE1. Learning how to use social networking websites for recruitment purposes is easy.
	EE2. The interaction of recruitment staff with social networking websites is clear and understandable.
	EE3. Recruitment staff finds social networking websites easy to use.
	EE4. It is easy to become skillful at using social networking websites for recruitment purposes.
Social influence Venkatesh et al. (2012)	SI1. People who are important to us think that we should use social networking websites for recruitment purposes.
	SI2. People who influence us think that we should use social networking websites for recruitment purposes.
Facilitating conditions Venkatesh et al. (2012)	FC1. Our organization has necessary financial resources to use social networking websites for recruitment purposes.
	FC2. Our recruitment staff has ample knowledge to use social networking websites for recruitment purposes.
	FC3. Our organization has necessary technological resources to use social networking websites for recruitment purposes.
	FC4. A technical assistance is available when we have difficulties in using social networking websites for recruitment purposes.
Hedonic motivation Venkatesh et al. (2012)	HM1. Using social networking websites for recruitment purposes is a kind of fun activity.
	HM2. Using social networking websites for recruitment purposes is enjoyable.
	HM3. Using social networking websites for recruitment purposes is very entertaining.
Price value Venkatesh et al. (2012)	PV1. Using social networking websites for recruitment purposes is reasonably priced.
	PV2. Using social networking websites for recruitment purposes is a good value for the money.
	PV3. At the current price, using social networking websites for recruitment purposes provides a good value.
Habit Venkatesh et al. (2012)	HT1. Using social networking websites for recruitment purposes can become a habit for recruitment staff.
	HT2. Using social networking websites for recruitment purposes can become an addiction for recruitment staff.
	HT3. Recruitment staff must use social networking websites for recruitment purposes.
SMART cost per hire Kashi (2015)	COST1: SNWs help our organization in reducing costs incurred to get a qualified candidate to accept an offer.
	COST2: SNWs help our organization in reducing the sourcing and marketing costs incurred to bring in the right candidate.
SMART Time to Fill Post Kashi (2015)	TIME1: SNWs help our organization in reducing the time span between the first advertisement of a vacancy and the acceptance of the offer by a job candidate.
	TIME2: SNWs help our organization in reaching the relevant candidates in a shorter amount of time.
	TIME3: SNWs help our organization zero-in faster on ideal job candidates.
SMART recruitment quality Kashi (2015)	RQ1: SNWs help our organization in expanding the job candidate reach beyond their personal networks.
	RQ2: SNWs help our organization in targeting the passive candidates who might not otherwise apply for the job vacancy advertised.
	RQ3: SNWs help our organization in better recruiting for positions that require certain skills.
	RQ4: The acceptance of social networking websites has helped our organization in attracting qualified and scarce candidates
	RQ5: SNWs help our organization in getting more information about job candidates to better assess them.
	RQ6: SNWs help our organization in targeting a specific job level to recruit.
Use of SMART Kashi (2015)	ACC1: Our HR staff use SNWs for searching for candidates
	ACC2: Our HR staff use SNWs for establishing initial contacts
	ACC3: Our HR staff use SNWs for background review and reference check
	ACC4: Our HR staff use SNWs for disseminating information
	ACC5: Our HR staff use SNWs for advertising job vacancies
	ACC6: Our HR staff use SNWs for developing professional networks
	ACC7: Our HR staff use SNWs for creating or maintaining a group or page on SNWs for the organization
Intention to adopt SMART Yoon and George (2013)	ITA1. Our organization intends to continue using social networking websites for recruitment purposes.
	ITA2. It is likely that our organization will find ways to improve the use of social networking websites for recruitment purposes.
	ITA3. It is likely that our organization will take further steps to better incorporate social networking websites to current recruitment methods.

### Analysis methods

The recruitment model proposed in the study was assessed using a two-stage structural equation modeling (SEM). Research suggests that SEM has a beneficial impact on assessing complex models such as models involving higher-order constructs and relationships involving mediation or moderation (MacKenzie et al., 2011; Hair et al., 2012). SEM analysis adopted in this study has a two-step approach involving a measurement model and a structural model analysis suggested by Anderson and Gerbing (1988). In the first step, the fit indices of the measurement model along with instrument validity and reliability were assessed, and in the second stage, structural model fit and relationship among variables were assessed to test the research hypotheses.

## Data analysis and results

### Construct reliability and validity

The reliability of measures used in the study was assessed with two essential values, i.e., Cronbach’s alpha values and composite reliability (CR) values. Nunnally (1978) highlighted that Cronbach’s alpha values and CR values should be greater than 0.70, and all values are within the range of cut-off values (Table 3). Fornell and Larcker (1981) recommended that average variance extracted (AVE) values should be greater than 0.50 for the assessment of convergent validity, and the values in Table 3 show the establishment of good convergent validity.

**TABLE 3:** Inter-correlation matrix, reliability of constructs, composite reliability, and average variance extracted.

Variables	Mean	CA	CR	AVE	1	2	3	4	5	6	7	8	9	10	11	12
1. Performance expectancy	4.09	0.875	0.87	0.637	**0.798**											
2. Effort expectancy	4.14	0.882	0.88	0.653	0.771	**0.808**										
3. Facilitating conditions	4.29	0.879	0.87	0.644	0.744	0.742	**0.802**									
4. Social influence	4.56	0.780	0.78	0.640	0.732	0.727	0.706	**0.800**								
5. Hedonic motivation	4.28	0.825	0.82	0.613	0.727	0.725	0.718	0.725	**0.783**							
6. Price value	4.41	0.836	0.83	0.629	0.718	0.701	0.702	0.729	0.705	**0.793**						
7. Habit	4.35	0.823	0.82	0.607	0.726	0.699	0.704	0.710	0.697	0.730	**0.779**					
8. Intention to adopt SMART	4.13	0.853	0.85	0.657	0.727	0.707	0.727	0.679	0.702	0.674	0.694	**0.811**				
9. Use of SMART	4.11	0.924	0.92	0.636	0.784	0.778	0.796	0.742	0.752	0.732	0.716	0.765	**0.798**			
10. SMART cost	4.29	0.808	0.81	0.681	0.644	0.617	0.669	0.649	0.624	0.647	0.625	0.645	0.683	**0.825**		
11. SMART time	4.26	0.896	0.89	0.742	0.664	0.633	0.695	0.660	0.611	0.610	0.636	0.642	0.682	0.744	**0.861**	
12. SMART recruitment quality	4.23	0.936	0.93	0.711	0.704	0.703	0.692	0.716	0.676	0.698	0.686	0.675	0.697	0.784	0.768	**0.843**

Correlation is significant at the 0.01 level.

### Discriminant validity analysis

For discriminant validity, the square roots of all AVE values of respective constructs were calculated and shown in the diagonal (bold values) of Table 3; all values were greater than the corresponding inter-correlation values, which concluded that the measurement model of the study has discriminant validity. In CFA, each indicator’s mean of items and scores were computed. To establish the measurement model, confirmatory factor analysis (CFA) was performed. The CFA results are shown in Table 4, which demonstrated that all values are significant and meet threshold values, also all model fit indices exhibited good model fit including χ2 = (1070.77), *p* < 0.01: NFI = 0.952; CFI = 0.989; IFI = 0.988; and RMSEA = 0.02.

**TABLE 4:** Measurement model.

**χ ^2^**	1070.77	**NFI**	0.952
**Df**	836	**CFI**	0.989
**χ ^2^/df**	1.28	**TLI**	0.988
**RMSEA**	0.02	**IFI**	0.989

### Assessment of the structural model and hypotheses testing

The structural model for the current study revealed a good model fit. The assessment of the structural model (Table 5) showed that the construed model fulfills the fit criteria; χ2 = (50.31), *p*°< 0.01: NFI = 0.993; CFI = 0.996; IFI = 0.996; RMSEA = 0.04.

**TABLE 5:** Structural model of the current study.

**χ ^2^**	50.31	**NFI**	0.993
**Df**	24	**CFI**	0.996
**χ ^2^/df**	2.09	**TLI**	0.990
**RMSEA**	0.04	**IFI**	0.996

**FIGURE 2:**
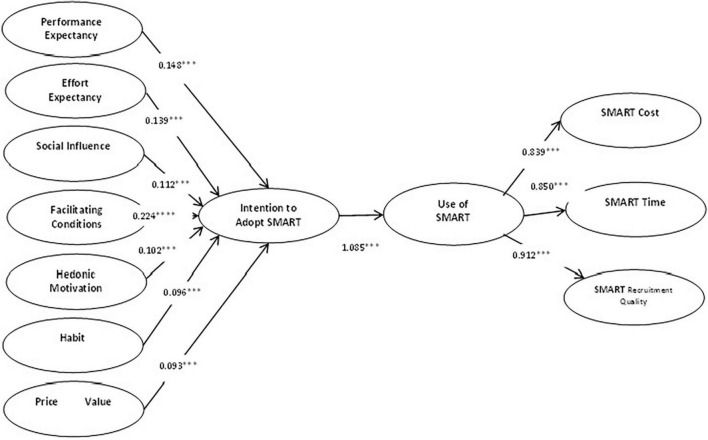
The structural model with path estimates.

The sign, size, and significance of the structural path coefficients and R2 values allowed an initial evaluation of the structural model. The standardized path coefficient (β) and *t*-values shown in Table 6 indicate that all the hypotheses were supported. The structural model gauged the testing of proposed hypotheses and calculated the fit of a model of recruitment through social media recruiting technology. Application of path estimates figure (Figure 2) explains the graphical alignment to determine the number of inclusive constructs and their influence over one another (Kline, 2011). In Table 6, path estimates are presented.

**TABLE 6:** Path estimates and testing of hypotheses.

Hypotheses	Paths	β	*T*
H1a	Performance expectancy-intention to adopt SMART	0.148***	5.60
H1b	Effort expectancy-intention to adopt SMART	0.139***	5.46
H1c	Social influence-intention to adopt SMART	0.112***	4.57
H1d	Facilitating conditions-intention to adopt SMART	0.224***	8.58
H1e	Hedonic motivation-intention to adopt SMART	0.102***	4.22
H1f	Habit-intention to adopt SMART	0.096***	3.92
H1g	Price value-intention to adopt SMART	0.093***	3.88
H2	Intention to adopt SMART-use of SMART	1.085***	29.43
H3	Use of SMART-SMART cost	0.839***	23.77
H4	Use of SMART-SMART time	0.850***	23.99
H5	Use of SMART-SMART recruitment quality	0.912***	25.80

***Significant at the 0.001 level.

Hypotheses 1a–g proposed that the performance expectancy, effort expectance, social influence, facilitating conditions, hedonic motivation, habit, and price value impact the intention to adopt SMART. Consistent with past research, the results revealed that performance expectancy has a significant positive impact on intention to adopt SMART (β = 0.148, *p* < 0.01) (H1a accepted). Similarly, the results indicated that effort expectancy (β = 0.139, *p* < 0.01) (H1b accepted), social influence (β = 0.112, *p* < 0.01) (H1c accepted), facilitating conditions (β = 0.224, *p* < 0.01) (H1d accepted), hedonic motivation (β = 0.102, *p* < 0.01) (H1e accepted), habit (β = 0.096, *p* < 0.01) (H1f accepted), and price value have a positive significant impact on intention to adopt SMART (β = 0.093, *p* < 0.01) (H1g accepted).

Hypothesis 2 proposed that the intention to SMART has a positive relationship with the use of SMART. Like previous literature, the results showed that the intention to SMART appeared to be positively related to the use of SMART (β = 1.085, *p* < 0.01), which supports H2.

Hypotheses 3–5 proposed that the use of SMART is significantly related to SMART cost, time, and recruitment quality. Consistent with previous research, the analysis revealed the simultaneous role of the use of SMART. The current study explained that the use of SMART significantly relates to SMART cost (β = 0.839, *p* < 0.01) (H3 accepted), SMART time (β = 0.850, *p* < 0.01) (H4 accepted), and SMART recruitment quality (β = 0.912, *p* < 0.01) (H5 accepted).

Path model disclosed that all proposed relationships were proved among performance expectancy, effort expectancy, social influence, facilitating conditions, hedonic motivation, habit, price value, intention to adopt SMART, use of SMART, SMART cost, SMART time, and SMART recruitment quality to be significant. Thus, all hypotheses of the study are accepted, and the model is significantly fit (Table 6) and establishes the recruitment model.

## Discussion

Organizations have realized the need and use of social networking websites (SNWs) for recruiting top and rare talent. SNWs have given a new face to recruitment trends. SNWs are considered an important recruitment tool though recruiting through social media technology is an ongoing credible area of concern for researchers and practitioners. We have discussed in the study how social media recruiting technology (SMART) can help in achieving desired recruiting outcomes of an organization for which the study used the UTAUT2 framework to explore and establish the empirical links between intention to adopt social media recruiting technology (SMART), acceptance of SMART, and the outcomes of SMART. This study proposed a conceptual model for recruitment. Acceptance of SMART significantly predicted SMART pre-hire outcomes such as cost, time, and recruitment quality. Such a comprehensive model for employee recruitment has never been discussed in earlier studies, through which organizations will get an opportunity to experience enhancement in the scope of recruitment as a whole instead of considering recruitment as a traditional procedure. The use of information technology can be expanded for progressive measures in employee recruitment. UTAUT2 framework has been used in the study to explore and establish the empirical links between intention to adopt SMART, use of SMART, and the outcomes of SMART. The extensive cross-sectional data of 589 manufacturing and service-oriented Pakistani organizations were collected to test the hypotheses and proposed research model.

*H1a*–*g* investigated the influence of different factors on the intention to adopt SMART. The findings revealed that performance expectancy, effort expectancy, social influence, facilitating conditions, hedonic motivation, and habit were strong predictors of the intention to adopt SMART. These findings are in line with the strong evidence in prior literature (Morosan and DeFranco, 2016; Baabdullah, 2018; Tamilmani et al., 2018), indicating that considering such factors can lead to the intention to adopt SMART. Moreover, these findings are also consistent with the components of the UTAUT2 model provided by Venkatesh et al. (2012), and the study narrated that all factors are significant and have a positive relationship with the intention to adopt SMART. As expected, outcomes of high performance and convenience are associated with social media recruiting technology considered to be the driving source to develop an individual’s intention to use technology; similar to the earlier studies (Kurfalı et al., 2017; Kemp et al., 2019), performance expectancy and effort expectancy have been evolved in this study as the most vital factors influencing the intention to utilize technological means. Findings of the study have also highlighted that if someone around is giving importance and significance to work or the use of technology, then there is a likelihood of the individual being more inclined toward the work and technology adoption (Rehman, 2018; Rehman et al., 2021). Another vital factor facilitating conditions is also proved to have a significant impact on the intention to adopt social media recruiting technology. Upon analyzing the results, a resemblance with earlier studies has been found (Oliveira et al., 2016; Macedo, 2017), and it has been observed that when the individual believes that infrastructure is in support of the use of the system, then he/she is more like to adopt the technology. Another critical factor influencing the intention to adopt social media recruiting technology is hedonic motivation, as it is associated with the fun side of using technology and considering work as enjoyment; therefore, it proved to be a positive influence on SMART adoption. An individual needs technology experience, but it is not enough to develop a habit; thus, it is developed when an individual explores and experiences technological features in multiple periods. Thus, the habit variable has been observed to have a positive relationship with social media recruiting technology adoption by considering the impact of previous learning experience and know-how about technology. The price value is the last factor described in UTAUT2, in line with the previous literature (Faqih and Jaradat, 2021; Tamilmani et al., 2021); it also proved to be associated with the tradeoff between benefits and cost for developing the intention of a consumer about use of technology. It is directly proportional to other financial benefits gained from the technology after successful adoption (Barba-Sanchez et al., 2018). Organizations do not consider technology necessary until or unless the provided benefits are improved efficiency, time-saving, and cost-effectiveness.

*H2* examined whether the intention to adopt SMART has a positive and significant relationship with the use of SMART, and the findings confirmed that the intention to adopt SMART positively relates to the use of SMART. This finding follows previous literature, which suggested that the intended behavior could be considered the motivational factor that may influence behavior and determines the potential indicators of an individual’s willingness and effort to engage in a particular act or behavior (Parry and Wilson, 2009; Li et al., 2021). Furthermore, this finding is in line with the study (Kashi, 2015; Olasina and Mutula, 2015). It is argued that the current study also sights the pre-hire SMART recruitment outcomes that ultimately fulfill the organization’s recruitment objective. This finding is consistent with Venkatesh et al. (2012) and Kashi (2015) that the insight about the use of SMART may help in gathering a pool of potential candidates by attracting, sourcing, and approaching them *via* social networking websites for a specific job role. It is highly likely that the intention to adopt social media recruiting technology could be the main factor that impacts the decision of an organization to utilize technologically innovative recruitment systems.

*H3–H5* investigated whether the use of SMART leads to SMART outcomes such as cost, time, and recruitment quality. The results corroborate support for these hypotheses like previous literature (Alsultanny and Alotaibi, 2015; Manuaba and Darma, 2021; Sengupta et al., 2021) because the use of SMART significantly predicted SMART outcomes such as cost, time, and recruitment quality. The literature on the use of SMART has suggested that aligning and devising a suitable recruitment process is similar to achieving recruitment goals (Breaugh, 2008). Prior literature suggests that the objectives or outcomes of recruitment can be further segregated into two dimensions, i.e., pre-hire and post-hire objectives (Breaugh, 2008, 2017; Kashi, 2015; Rehman, 2018; Pessach et al., 2020). These findings differentiate the study from previous research that has pondered over multiple features of post-hire outcomes, whereas this study targeted the aspects of pre-hire outcomes where the amount of information and research on technological use is little. The current study highlighted three outcomes of the use of SMART highlighted through literature, i.e., SMART Cost, SMART Time, and SMART Recruitment Quality for pre-hire objectives. The findings of the current research indicated that the SMART cost, the SMART time, and the SMART recruitment quality could be improved through the use of SMART. These findings could be considered the unique contribution of the current study. Such a comprehensive model has never been discussed in earlier studies and opened the door to innovation to meet global technology innovation challenges, especially in the new normal era after the pandemic. Thus, the current research supported that the pre-hire outcomes could be improved after the use of social media recruiting technology in the recruitment process.

## Study implications

This study has highlighted significant theoretical and practical implications for researchers, SNWs users, employee recruiters, and policymakers, which are as follows:

### Theoretical implications

The current study has two key theoretical implications. *First*, the current study has analyzed the theoretical foundation of social media recruiting technology (SMART), which gives reason to organizations to accept or reject the technology. In contrast, the conceptual model with factors description in the context of empirical validation has been examined along with different SMART outcomes. The primary purpose of this research was to highlight the use of social media and information technology in employee recruitment by providing a conceptual recruitment model in the aspects of theoretical foundations and empirical evidence on the factors and outcomes leading to the use of social media recruiting technology. The results showed that the model of social media recruiting technology for employee recruitment had been validated in the contextual framework of Pakistan to extend the body of current knowledge. The conceptual model of this study has integrated the Extended Unified Theory of Acceptance and Use of Technology (UTAUT2) in the aspect of the use of SMART and for the evaluation of pre-hire outcomes of SMART in an organizational setting which can be considered a positive step toward sustainable development. This recruitment model can further serve other developing and developed nations with less difference in a contextual setting.

*Second*, the current research has theoretically highlighted the factors of the Extended Unified Theory of Acceptance and Use of Technology (UTAUT2) as influencing variables for the use of SMART and SMART cost, time, and recruitment quality as outcome variables in the context of employee recruitment. This study has validated the revision of UTAUT2 and extension in the theory of employee recruitment through technological innovations. This study proposed a framework of SMART from the perspective of Pakistani organizations to minimize the gap in theoretical and empirical knowledge. One significant dynamic in the extension of SMART is the critical aspect of staffing due to global competitiveness. Therefore, organizations need to accept SMART to attract and retain potential and qualified candidates for sustainability.

### Practical implications

It has become a need of the day that all organizations should incorporate the latest employee recruitment trends to keep up with modern drifts. The inclusion and integration of SNWs in businesses have modified the recruiting trends and improved the recruitment quality with lower cost and optimum recruiting time with a parallel contribution to achieving innovation. Besides the benefits in terms of cost, time, and quality, SNWs are a more significant source to target a more extensive and relevant pool of audiences. In some ways, recruitment through SNWs is the contemporary form of E-recruitment program that encourages organizations to enrich their hiring decisions by targeting and ensuring a pool of potential candidates.

The current research has two significant practical implications for recruiters and policymakers. *First*, the current study will guide professionals in effectively using social networking websites (SNWs) and platforms. SNWs help organizations win the battle of acquiring human capital through less time and money consumption with quality candidates. HR and recruitment managers can use SMART positively and further explore and gather information about candidates to make the recruitment process more effective. Furthermore, the managers may accomplish their strategies to group the potential ones from the pool of entrants. The study substantially contributes to human resources, innovation management, and information technology. It opens a new platform to improve the strategies compared to traditional recruitment tactics and safeguards the interest of both organizations and candidates.

*Second*, the current study revealed several recruitment outcomes. It emphasized the applications of social media recruiting technology by the adjacent bunch of factors, intention to adopt SMART, use of SMART, and the outcomes of SMART. Meanwhile, the study proved how the recruitment process might be uplifted through the use of SMART to scrutinize higher-quality entrants. The study also proposed a mechanism that curtails the proceeding time of employee recruitment and also lightened the lesser associated cost with the adulation of a comprehensive model that has not been studied so far. The outcomes of the study highlighted the importance of intention to adopt SMART and the use of SMART endorsed by the organizations and HR managers, keeping in view the benefits of SMART outcomes regarding pre-hire objectives. The pre-hire objectives incurred as the consequence of the use of SMART and further completion of the recruitment process suggested by Breaugh (2008) include scrutinizing the quality candidates for concerned positions, the shortened time of the recruitment process, and lesser proceeding cost with the achievement of sustainability objectives at the organizational level.

## Limitations and future work

Besides the contribution, the current study shares some common limitations, which may arise the need for future research. *First*, the current study has grouped the data set by switching the organizations registered on the Pakistan Stock Exchange and Lahore Chamber of Commerce, Pakistan. For a broader understanding, current phenomena may be stretched by keeping sight of the organizations registered at other chambers of Pakistan and different countries. For replication of the study, a comparative study can also be performed. *Second*, the considerable segment of respondents was men, with a percent of 84, which may provoke gender nepotism. So, this SMART model should be evaluated in countries where men and women work in equal ratios. In the future, the current model could be deployed by other constructs of adjacent nature like more technological factors or by framing some personality-related factors of recruiting managers. *Third*, the current study did not put the quantum of business age, size, and nature under their areas of concern, which can be moderated in future studies. Future researchers are encouraged to build acquainted relations with such factors by developing a mechanism for their assimilation. *Fourth*, this study has worked on self-reported data but multi-layer studies should be conducted with this comprehensive model in the future for replication.

## Data availability statement

The raw data supporting the conclusions of this article will be made available by the authors, without undue reservation.

## Ethics statement

Ethical review and approval was not required for the study on human participants in accordance with the local legislation and institutional requirements. Written informed consent from the patients/participants or patients/participants legal guardian/next of kin was not required to participate in this study in accordance with the national legislation and the institutional requirements.

## Author contributions

SR presented main theme and worked on overall analysis and methodology. AU worked on introduction and data collection. KN worked on literature review. AE worked on discussion. HE worked on proofreading and implications. All authors contributed to the article and approved the submitted version.
